# Agro-Industrial Waste Valorization for Sustainable PHBV Production from Sugarcane Bagasse Using *Bacillus* sp. HLI02

**DOI:** 10.3390/polym18070802

**Published:** 2026-03-26

**Authors:** Komal Singh, Preeti Tomer, Debarati Paul, Narayan Chandra Mishra, Tanushri Mukherjee, Debashish Ghosh, Monica Trif, Sourish Bhattacharya, Alexandru Vasile Rusu, Saugata Hazra

**Affiliations:** 1 Department of Bioscience & Bioengineering, Indian Institute of Technology, Roorkee 247667, India; komal_s@pe.iitr.ac.in (K.S.); preetitomer7@gmail.com (P.T.); 2 CSIR-Central Salt and Marine Chemicals Research Institute, Bhavnagar 364002, India; pauldebarati27@gmail.com (D.P.); tanumukherji06@gmail.com (T.M.); sourishb.csmcri@csir.res.in (S.B.); 3 Department of Polymer and Process Engineering, Indian Institute of Technology, Roorkee 247667, India; narayan.mishra@pe.iitr.ac.in; 4 Material Resource Efficiency Division, CSIR-Indian Institute of Petroleum, Mohkampur, Dehradun 248005, India; 5 Department of Food Science, University of Agricultural Sciences and Veterinary Medicine Cluj-Napoca, Manastur 3–5, 400372 Cluj-Napoca, Romania; monica_trif@hotmail.com; 6 CENCIRA Agrofood Research and Innovation Centre, Ion Meșter 6, 400650 Cluj-Napoca, Romania; 7 Centre for Nanotechnology, Indian Institute of Technology Roorkee, Roorkee 247667, India

**Keywords:** agro-industrial waste, polyhydroxyalkanoates, *Bacillus*, valorization, sugarcane bagasse waste, packaging, coating, bioeconomy

## Abstract

The large-scale production of microbial bioplastics remains limited by high production costs, reliance on refined substrates, and inefficient utilization of agro-industrial residues. Although sugarcane bagasse has been explored as a carbon source for polyhydroxyalkanoate production, studies have predominantly focused on poly (3-hydroxybutyrate) (PHB), with limited reports on copolymer synthesis from pentose-rich lignocellulosic streams. In this study, a newly isolated *Bacillus* sp. HLI02 was employed for the biosynthesis of poly(3-hydroxybutyrate-co-3-hydroxyvalerate) (PHBV), using pentosan-rich sugarcane bagasse hydrolysate as an inexpensive and sustainable carbon source. Fermentation parameters were systematically optimized at different pH and temperature, and the strain demonstrated efficient conversion of xylose-rich hydrolysate into PHBV without the requirement for external nutrient supplementation. Under optimized conditions (pH 7.0, 37 °C, and C/N ratio of 40), a maximum PHBV yield of 2 g/L, corresponding to 59.5% of cell dry weight, was achieved. Structural and compositional analyses using Fourier transform infrared spectroscopy (FTIR), gel permeation chromatography (GPC), and ^1^H and ^13^C nuclear magnetic resonance (NMR) spectroscopy confirmed successful PHBV copolymer formation with well-defined structural characteristics. Thermal analysis revealed a decomposition temperature of 166 °C, indicating good thermal stability. The produced PHBV further exhibited favourable biocompatibility and biodegradability, supporting its potential applicability in sustainable packaging and related sectors. This work demonstrates the effective conversion of hemicellulosic sugarcane bagasse hydrolysate into PHBV using a newly isolated *Bacillus* strain, highlighting an underexplored route for copolymer production from agro-waste–derived C5 sugars. By integrating low-cost feedstock utilization with process optimization and comprehensive polymer characterization, this study contributes to the development of economically viable and sustainable bio-based polymer production strategies.

## 1. Introduction

Petrochemical-based plastics are predominantly non-biodegradable, contributing significantly to environmental pollution and posing long-term risks to human health [1,2]. The increasing demand for sustainable materials has prompted extensive research into environmentally friendly alternatives, with bio-based polymers emerging as a promising solution. Poly(3-hydroxybutyrate-co-3-hydroxyvalerate) (PHBV) exhibits physicochemical properties comparable to petroleum-based plastics, including high tensile strength, thermoplastic processability, good oxygen and moisture barrier characteristics, and tunable flexibility depending on hydroxyvalerate content. In addition, PHBV possesses excellent biodegradability under soil and marine conditions, biocompatibility, and thermal stability suitable for conventional melt-processing techniques, making it a promising sustainable alternative to polypropylene and related commodity plastics. Microorganisms naturally produce polyhydroxyalkanoates (PHAs), which are a renewable, biodegradable, and environmentally acceptable substitute for traditional petroleum-based plastics with potential uses in the biomedical, agricultural, and sustainable packaging industries. [3,4,5,6,7,8].

Despite these advantages, large-scale PHA production remains limited by high manufacturing costs compared with petrochemical plastics. Strategies to improve economic feasibility include the selection of high-yield PHA-accumulating microorganisms, utilization of low-cost or waste-derived carbon sources, optimization of culture conditions, and improved downstream recovery methods [9,10,11,12]. Among biodegradable polymers, fully degradable bioplastics such as polylactides (PLA), thermoplastic starch (TPS), and PHAs have gained prominence [13,14]. PHAs are highly versatile, and more than 300 bacterial strains, including *Bacillus*, *Halomonas*, and *Pseudomonas*, are known to synthesize them [15,16,17]. *Ralstonia eutropha*, now classified as *Cupriavidus necator*, is one of the most thoroughly studied and industrially important bacteria for polyhydroxyalkanoate (PHA) synthesis due to its ability to accumulate PHA up to 80–90% of its cell dry weight under nutrient-limited circumstances. The strain has been used as a model organism for large-scale PHA fermentation and metabolic engineering experiments due to its broad substrate flexibility, effectively using sugars, fatty acids, plant oils, and lignocellulosic hydrolysates [18,19,20].

**Figure 1:**
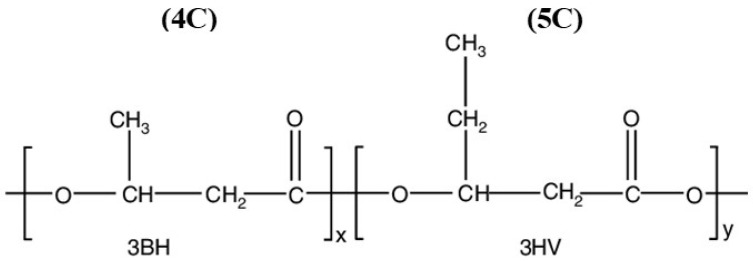
Chemical structure of PHBV copolymer.

PHAs demonstrate broad applicability across sustainable packaging and other sectors. They are used in food packaging, biodegradable containers, fibres, eco-bags, and medical devices, such as sutures and drug delivery systems [21,22,23,24]. They can also serve as biofuels via conversion to hydroxyl-alkanoate methyl esters (HAMEs), enhancing ethanol combustion [25,26]. Poly(3-hydroxybutyrate-co-3-hydroxyvalerate) (PHBV) has better mechanical flexibility, lower brittleness, lower crystallinity, and better thermal processability than poly(3-hydroxybutyrate) (PHB). The addition of hydroxyvalerate units disrupts PHB’s highly crystalline structure, resulting in increased elongation at break, broader processing temperature window, and better toughness. Because of these benefits, PHBV (Figure 1) is preferable to PHB in packaging and biomedical applications where durability and flexibility are crucial [7,27,28].

Recent studies increasingly focus on cost-effective PHBV production using *Bacillus* species due to their robustness, versatility, and ability to utilize agro-industrial residues as carbon sources. Sugarcane bagasse is one such attractive lignocellulosic feedstock, abundant and inexpensive, composed of approximately 40–55% cellulose, 20–30% hemicellulose, and 18–24% lignin [29,30,31]. During dilute-acid pretreatment, hemicellulose is preferentially hydrolyzed, releasing soluble pentose sugars such as xylose and arabinose, while most of the crystalline cellulose and lignin remain enriched in the residual solid fraction, depending on pretreatment severity. Consequently, the resulting hydrolysate primarily contains fermentable C5 sugars, typically accompanied by minor amounts of C6 sugars, acetic acid released from acetylated hemicellulose, and trace levels of degradation products such as furfural and 5-hydroxymethylfurfural (HMF). The liquid fraction is therefore characterized by a high carbon content and a favourable C/N ratio, which supports microbial fermentation, although partial detoxification may be required to mitigate inhibitory compounds formed during pretreatment. Despite these favourable characteristics, most existing studies emphasize PHB production and focus predominantly on overall polymer yield, with comparatively few reports demonstrating PHBV biosynthesis, particularly from pentose-rich (C5) sugar hydrolysates derived from bagasse hemicellulose [32,33,34]. This limitation is largely attributed to the insufficient intracellular availability of propionyl-CoA precursors necessary for 3-hydroxyvalerate (3HV) monomer incorporation when pentose substrates are used without external precursor supplementation. Despite the ability of many *Bacillus* species to metabolize both pentose and hexose sugars, efficient microbial conversion of xylose-rich lignocellulosic hydrolysates into PHBV remains underexplored. In this context, the present study introduces a novel integrated bioprocess that combines sugarcane bagasse hydrolysate conversion, optimized strain selection (*Bacillus* sp. HLI02), and process optimization to produce high-yield, structurally and thermally robust PHBV. Both upstream (feedstock valorization) and downstream (material characterization) aspects were addressed, and therefore, this work highlights the potential of PHBV as a sustainable packaging material with suitable biodegradability, mechanical performance, and environmental compatibility. The present approach aligns with circular bioeconomy principles by valorizing sugarcane bagasse, an abundant agro-industrial residue, into high-value biodegradable PHBV, thereby enabling waste-to-wealth conversion and reducing dependence on fossil-derived plastics. Furthermore, the use of inexpensive lignocellulosic feedstock, pentose-based fermentation without external valerate supplementation, mild pretreatment conditions, and a robust *Bacillus* strain capable of stable polymer production collectively enhance the techno-economic feasibility and commercial scalability of the proposed bioprocess. The approach demonstrates a clear advancement over conventional PHA production systems, emphasizing circular bioeconomy principles, commercial viability, and application-driven polymer development.

## 2. Materials and Methods

### 2.1. Sample Collection and Isolation of PHA-Producing Bacteria

*Bacillus* sp. HLI02 was isolated from rhizospheric soil collected from agricultural land in Odisha, India. The isolate was cultivated in modified minimal salt medium (MSM) and Zobell marine broth (ZMB) for 72 h to induce PHA accumulation. Preliminary screening of PHA-producing bacteria was conducted by Sudan black staining, as it confirms the presence of intracellular PHA. 0.3 mg of Sudan black B powder dye was dissolved in 100 mL of 70% ethanol. The bottle containing the solution was shaken for almost 5 min and filtered into a fresh bottle. A smear of bacteria has been created on a clean glass slide and heat fixed on the glass slide. The smear was stained with Sudan black and incubated for 10 min. The slides were washed and air-dried. The cells were observed under 1000X, Leica DM5000B light microscope (Tokyo, Japan). The positive Sudan black-stained isolates were streaked on starch agar plates and cultured for 24 h at 30 °C for the formation of pure colonies. After incubation, the colonies were further confirmed by Sudan black staining under a microscope, where blue-black or violet intracellular granules indicate PHB granule accumulation [35,36]. Further, for confirmation of screening, pure isolates were grown on NL-M agar supplemented with 2% glucose and trace elements, containing 0.5 μg/mL of Nile Blue staining dye. Plates were exposed to UV radiation (260–300 nm) followed by a 48 h incubation period at 37 °C. After that, the colonies observed under an Olympus 1 × 71 fluorescence microscope (Tokyo, Japan) produced an orange colour, indicating the synthesis of PHA polymer [37,38]. All biochemical and enzymatic experiments were undertaken in triplicate. *Escherichia coli* ATCC 25922 and *Bacillus subtilis* ATCC 6633 were employed as negative and positive reference strains, respectively, to validate test performance. All the chemicals, solvents, and reagents used in the study were of analytical grade and obtained from Sigma (Germany) unless mentioned otherwise.

### 2.2. Biochemical Characterization

The microscopic measurements were carried out to study the morphological features of *Bacillus* sp. HLI02. Endospore staining, Catalase and H2S production from cysteine, Indole, Methyl red test, Voges Proskauer, Ammonia Production, Nitrate reduction, Urease production, Citrate utilization, Oxidase, Mannitol, Esculin hydrolysis, Anaerobic growth, Blood Haemolysis, enzymatic such as (Gelatinase, Casein hydrolysis, Tributyrin, Amylase, Cellulase, Chitin hydrolysis, Pectin hydrolysis, DNase, Lecithinase) were performed as per the reported protocols [39]. The sugar utilization was evaluated by using various sugars, viz., xylose, mannose, maltose, sucrose, raffinose, dextrose, trehalose, fructose, glucose, ribose, lactose, rhamnose, esculin, inulin, mannitol, arabinose, sorbitol, and melibiose in the defined medium for 48 h at 37 °C [40].

### 2.3. Preparation of Production Media

Modified Minimal Salt Media (MSM) and Zobell Marine Broth (ZMB) were prepared for the growth and subsequent production of the polymer. The composition of the media is mentioned in Table 1. Control MSM was prepared with xylose, which was subsequently replaced by sugarcane bagasse. Xylose was selected not because the strain cannot utilize glucose, but because sugarcane bagasse hydrolysate contains majorly xylose and utilizing pentose is an essential scientific problem addressed in this study. Even though the isolated strain could use both pentose (C5) and hexose (C6) sugars mentioned in Table 2 xylose was chosen on purpose as the model carbon source for the growth of bacteria for PHB production. This choice was made since the aim of the study is to valorize the hemicellulose fraction of sugarcane bagasse, which predominantly releases pentose sugar, particularly xylose, during dilute acid hydrolysis. Xylose is the main fermentable sugar in bagasse hydrolysate; therefore, using it made it possible to accurately simulate the actual substrate composition and test the strain’s capacity to turn C5 sugars into value-added biopolymers. The pH was maintained by adding 0.1 N H_2_SO_4_ and 0.1 N NaOH, and then was autoclaved for sterilization.

### 2.4. Substrate Preparation/Waste Collection and Hydrolysate Preparation

Sugarcane bagasse, a fibrous solid waste of sugarcane after juice extraction, was collected in bulk from the nearby farmland. The collected waste was first washed with water and dried in the air under shade. Then, dried biomass was ground in a mixer grinder to get the fine powder of particle size around 3–4 mm. Further, the fine powder was digested with diluted H_2_SO_4_ (0.25% *v*/*v*) in a custom-made biomass digester. For the digestion, the solid-to-liquid ratio was maintained at 1:10 at 140 °C for around 1.5 h under autogenous pressure conditions. The pretreatment, with diluted H_2_SO_4_, was employed for the hydrolysis of lignocellulosic content present in the biomass, to recover most of the hemicellulose-derived sugars of interest. In the present work, for the recovery of C5 (pentose) sugar, sugarcane bagasse was utilized as lignocellulosic feedstock. After pretreatment, the resulting liquid fraction was over-limed to facilitate clarification and then used as a pentosan-rich carbon source for biopolymer production. Sugarcane bagasse is a well-known biomass material, mainly composed of cellulose, hemicellulose, and lignin. From previously reported studies, its composition typically includes cellulose (40–55%), hemicellulose (20–30%), and lignin (18–24%). Under mild conditions, pretreatment of hemicellulose results in the release of soluble pentose sugars such as xylose and arabinose, whereas cellulose and lignin are unaltered. A compositional analysis before and after pretreatment is beyond the scope of this work; hence, the literature-based values were used to describe the substrate characteristics. The over-liming treatment favoured the precipitation of soluble lignin, phenolic compounds, and furans, yielding a clarified hydrolysate with a high concentration of pentose sugars. The chemical composition of the sugarcane bagasse hydrolysate, including quantitative profiles of pentose content such as xylose concentration and recovery efficiency, was determined by following the procedure described in a previous study [41]. High-performance liquid chromatography (HPLC) was utilized for sugar analysis. The composition of the bagasse hydrolysate displayed the presence of xylose as the main sugar, with minor components of arabinose, which are characteristic of the hemicellulosic composition of sugarcane bagasse. Although arabinose was identified as a minor component of pentose sugars, its concentration was significantly lower than that of xylose. Therefore, the main fermentable sugar was identified as xylose, which was the main carbon source for the biosynthesis of PHBV. The capacity of *Bacillus* sp. HLI02 to metabolize arabinose was confirmed in the sugar utilization test, and therefore, the minor concentration of arabinose in the hydrolysate could be used as a carbon source for the biosynthesis of PHBV during the fermentation process.

### 2.5. Optimization of Process Parameters for Polymer Production

The strain was cultured in batch fermentation under controlled conditions. Key parameters—including temperature, pH, agitation speed, and hydrolysate concentration—were optimized to achieve maximum polymer yield. Based on preliminary observations, *Bacillus* sp. HLI02 was selected for further investigation and cultivated in nitrogen-limiting minimal salt medium (MSM). The effects of temperature (16 °C, 23 °C, 30 °C, 37 °C, 44 °C, and 51 °C), pH (6, 7, 8, 9, 10, 11, and 12), and xylose concentration (5, 10, 15, 20, and 25 g/L) were evaluated over 72 h in an orbital shaker at 125 rpm to determine conditions that support maximum polymer production. The optimized fermentation conditions for *Bacillus* sp. HLI02 was established at 30 °C with agitation at 220 rpm for 30 h. Upon completion of fermentation, the cell biomass was harvested by centrifugation at 10,000 rpm for 15 min. The resulting cell pellet was dried at 60 °C prior to polymer extraction. Fermentation parameters were further refined using a one-factor-at-a-time (OFAT) approach, in which a single variable was modified while all other conditions were maintained constant. This method primarily focused on temperature and pH, as these are critical physiological parameters influencing microbial metabolism, enzymatic activity, and PHBV production. Other operational parameters—such as incubation time, agitation speed, inoculum size, and carbon source concentration—were maintained within previously reported optimal ranges for Bacillus-mediated PHA production. This method mainly emphasized pH and temperature values, as these are well-known physiological parameters that affect microbial metabolism and PHBV biosynthesis. Other operational conditions, such as inoculum volume concentration (5% *v*/*v*), agitation rate (220 rpm), and incubation period (30 h), were kept under consideration based on previously reported optimal values for Bacillus-mediated PHA biosynthesis. These conditions were kept constant during OFAT optimization. With the newly identified strain, *Bacillus* sp. HLI02, the OFAT strategy allowed for efficient preliminary screening and the determination of baseline operational parameters. Although the strategy does not account for possible interaction effects among the variables, it is still widely used in the early stages of strain characterization prior to using multivariate optimization techniques.

### 2.6. Extraction of Polymer Using Waste Resources

Extraction of the polymer was finally carried out using the chemical cell disruption technique. The obtained cell pellet was suspended in 40% sodium hypochlorite for 30 min for complete digestion of non-PHA materials (NPM). The minimum concentration of sodium hypochlorite for polymer extraction was determined using variable concentrations of hypochlorite in order to optimize a low-cost process. The resulting mixture was purified with distilled water, followed by acetone, methanol, and diethyl ether in a 1:1:1 ratio [42]. The extracted polymer was re-dissolved in chloroform and finally evaporated to yield a dry film of polymer.

The weight of the film was then calculated by the following Equation (1) and used for further characterizations:

Polymer production (%) = (Weight of polymer/Weight of biomass) × 100(1)

### 2.7. Nuclear Magnetic Resonance (NMR)

The ^1^H and ^13^C NMR spectroscopic measurements were carried out to recognize the chemical structure and functional group distribution. All spectra were collected using the JEOL JNM-LA NMR spectrometer of 500 MHz (USA). The samples were prepared by dissolving the polymer in deuteron chloroform (CDCl3) at a 2 mg/mL concentration. The solution of polymer in the CDCl3 was recorded with a 5 ms pulse width for 32,000 data points and 32 accumulations. The internal chemical shift standard used was Tetramethylsilane (TMS).

### 2.8. Fourier Transform Infrared Spectroscopy (FTIR)

The Fourier confirmed the presence of various functional groups in the polymer transform infrared (FTIR) spectroscopic analysis using a Perkin-Elmer RX 1 FTIR spectrometer. FTIR spectrum was obtained in the 4000–400 cm^−1^ range with a uniform thin film of PHA, prepared on a KBr pellet.

### 2.9. X-Ray Diffraction (XRD)

The phase study of the extracted polymer was carried out using a Powdered X-Ray Diffractometer (Bruker D8-Advance, Germany) with a 2θ range of 5° to 90°and a scan rate of 1°/min using Cu-Kα (λ = 1.5406 Å) radiation. It was operated at 40 kV and 30 mA.

### 2.10. Thermogravimetric Analysis (TGA)

Thermal analysis of extracted polymer was carried out using a Thermogravimetric Analyzer (EXSTAR, SII 6300 EXSTAR, Canada). It was performed with 4 mg of the sample at 30 °C to 700 °C at a heating rate of 10 °C/min under a liquid nitrogen atmosphere.

### 2.11. Gas Permeation Chromatography (GPC)

Molecular weight analysis of the polymer was done by Gel Permeation Chromatography (GPC) (Agilent GPC-Addon Rev B.01.0, USA) using a refractive index detector. The polydispersity index and average molecular weight were determined using the software. Chloroform (CHCl_3_) and Polystyrene (conc. 1.00 g/L) were used as molecular weight standard and mobile phase, respectively, with a flow rate of 0.3 mL/min.

### 2.12. Surface Characterization of Polymer

The surface morphology and topographical features of the polymer thin film were analyzed using Field Emission Scanning Electron Microscopy (FESEM) (QUANTA 200 FEG, FEI, The Netherlands) and Atomic Force Microscopy (AFM) (NT-MDT INTEGRA, scanning probe microscope). AFM, as a surface probe microscopy (SPM) technique, was employed to generate three-dimensional surface profiles and evaluate surface roughness parameters of the polymer thin film.

The extracted PHBV polymer was dissolved in chloroform to obtain a homogeneous polymer solution (1–2% *w*/*v*). The solution was filtered to remove any insoluble impurities and cast onto clean glass coverslips (for FESEM) and freshly cleaved mica substrates (for AFM analysis). Solvent evaporation was carried out at ambient conditions to allow gradual film formation. The resulting thin films were further dried under vacuum to ensure complete removal of residual solvent before surface characterization.

### 2.13. Biocompatibility of Polymer

The cytotoxicity of the polymer was checked on NIH/3T3 mouse fibroblast cells (ATCC CRL 1658) through viability (MTT) assay. Cells were grown in Dulbecco’s Modified Eagle Medium containing 1.5 g/L glucose (DMEM-Low Glucose, HiMedia, India), added with 10% fetal bovine serum (FBS) (Invitrogen, Carlsbad, CA, USA) and 1% antibiotic (100 U/mL of penicillin and 100 µg/mL streptomycin; from Hi-Media, India) in the incubation was maintained at 37 °C with 5% CO_2_ and 95% RHS. The previously preserved polymer sample was again washed with chloroform (2 gm of preserved sample/10 mL of chloroform) for purification. The purified polymer was dissolved in chloroform to make a 1 mg/mL stock solution. 10, 20, 30 and 40 μL of stock solution was applied on separate wells of a 96-well cell culture plate to coat the bottoms of the wells with final polymer concentrations of 5, 10, 20, 30 and 50 μg/mL volumes of the polymer stock used to coat the bottoms of the wells were calculated concerning the total assay volume, which was 50 μg/mL. The coating was dried at room temperature for 3–4 h to remove the chloroform, followed by complete UV irradiation overnight. Subsequently, 2 × 10^3^ cells/well were seeded to check the viability after 72 h. Cells grown in uncoated wells were taken as a control. Coated wells without cells were considered as corresponding blanks for each polymer concentration. 3-(4,5-dimethylthiazol-2-yl)-2,5 diphenyltetrazolium bromide (MTT) assay was carried out by adding 10 μL of 10 mg/mL MTT reagent to each well and incubating for 4 h at 37 °C. Formazan crystals thus formed were dissolved in 200 μL DMSO, and the optical density (OD) of the resulting purple solution was measured at 570 nm in the ELISA plate reader (Fluostar Optima, BMG Labtech, Germany), and it was used to calculate percentage cell survival.

### 2.14. Biodegradability of Polymer

The biodegradability test of the obtained polymers was carried out by the open window composting method. A pre-weighed polymer disc was incubated in the garden soil at 10 cm depth (temperature ~32 °C and pH 7.8). The discs were visually checked for changes in their morphology and weight loss at different time intervals of 20, 40, and 60 days [42]. After 20 and 40 days of incubation, the resultant polymer was washed with PBS (Phosphate-Buffered Saline), vacuum dried, and then weighed to measure weight loss. The microscopic changes in the polymeric samples were probed by capturing the FESEM images.

### 2.15. Statistical Analysis

All experiments were performed in triplicate unless otherwise stated. Results are expressed as mean ± standard deviation (SD). Statistical analysis was conducted using GraphPad Prism (GraphPad Software, USA). Differences between experimental groups were evaluated using one-way analysis of variance (ANOVA), followed by Tukey’s post hoc test. A *p*-value of less than 0.05 was considered statistically significant.

## 3. Results

### 3.1. Microorganism and Its Identification

**Figure 2:**
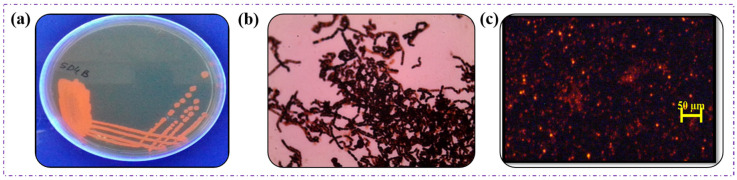
Screening of *Bacillus* sp. HLI02 under light microscope (**a**) Nile red staining, (**b**) Sudan black B staining and (**c**) Nile blue staining.

**Figure 3:**
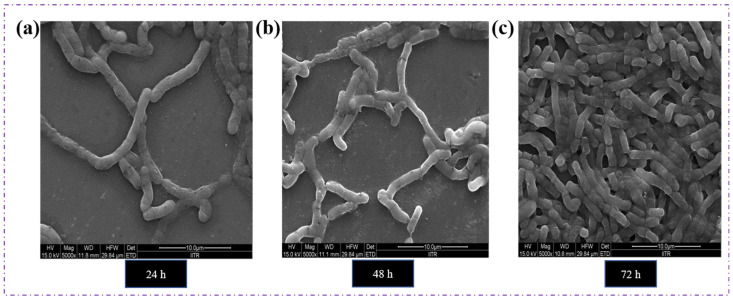
Screening of *Bacillus* sp. HLI02 under Fe-SEM. Change in the morphology of the cells at different time intervals due to polymer accumulation in the cell cytosol. 24 h, rod-shaped cells (**a**), 48 h, (**b**) intermediate stage, and 72 h (**c**) oval-shaped cells.

The *Bacillus* sp. HLI02 was successfully cultivated in Nutrient Broth and then transferred to MSM containing Nile red. After 72 h of incubation, the isolates were showing excellent light emission (Figure 2a). Similarly, the heavy bluish-black deposition in the bacterial cell after the Sudan black B staining (Figure 2b), and yellowish orange fluorescence (Figure 2c), Nile Blue A. Screening revealed the accumulation of polymer in the cell cytosol of bacteria. These three staining methods [43] have proven that the selected isolate is an excellent candidate for polymer production. The SEM images (Figure 3) explicitly demonstrate the gradual change in bacterial shape from rod to oval as a function of time, and it was associated with the accumulation of polymer granules. These observations indicate that polymer accumulation begins during early growth, and progressive granule enlargement at later stages likely induces the transition from rod-shaped to oval cells [44].

The biochemical, enzymatic, and sugar utilization characteristics have confirmed that the polymer-producing isolate HLI02 is a member of the genus *Bacillus*, as suggested by the standard microbiological characterization studies (Table 2 and Table 3). Although *Bacillus* sp. HLI02 can utilize several C5 and C6 sugars such as arabinose, glucose, fructose, maltose, and raffinose; the fermentation studies were designed to assess the utilization of pentose-rich substrates. Xylose is the major sugar present in sugarcane bagasse hydrolysate, whereas arabinose is present in amounts less than 10–15% of the total pentose. Consequently, the contribution of arabinose to the overall polymer synthesis is expected to be less significant; however, its assimilation may take place simultaneously with the fermentation process without affecting the PHBV synthesis. The capacity of the strain to utilize both pentose (C5) and hexose (C6) sugars is an indicator of the metabolic flexibility of the strain. Efficient conversion of xylose into PHBV is particularly relevant for lignocellulosic biorefineries, where pentose sugars are predominant but less effectively utilized by microbes. The efficiency of the production of PHBV from different sugars, including glucose and arabinose individually and in combinations will be studied in future research.

Phylogenetic analysis based on 16S rRNA sequencing further revealed a close relationship between HLI02 and *Bacillus endophyticus*, supported by secondary structural predictions illustrated in Supplementary Figures S1 and S2. These findings are in line with previous reports highlighting the prevalence of *Bacillus* species in diverse environmental niches, including rhizospheric soils and sludge, where they exhibit versatile metabolic capabilities and the potential for polyhydroxyalkanoate production [44]. The close genetic relationship with *B. endophyticus* suggests that HLI02 may share similar metabolic pathways for polymer biosynthesis, particularly the efficient utilization of pentose-rich sugarcane bagasse hydrolysate. This taxonomic and functional characterization not only validates the identity of HLI02 but also reinforces its suitability as a microbial platform for high-yield PHBV production, highlighting the potential for strain-specific optimization in agro-waste valorization strategies.

### 3.2. Optimization Parameters for Polymer Production

**Figure 4:**
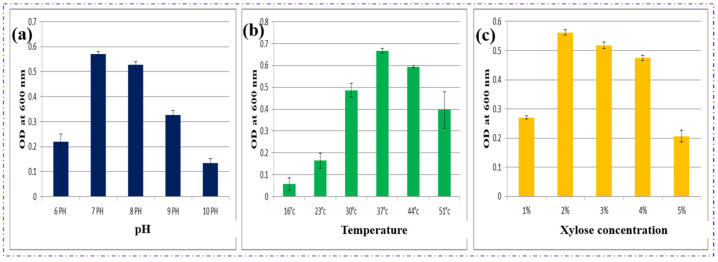
Optimization of various growth parameters of *Bacillus* sp. HLI02 on the basis of (**a**) pH, (**b**) temperature, and (**c**) xylose concentration. Data represent mean ± SD (n = 3).

The optimization of physicochemical and nutritional parameters is a critical step for maximizing polymer accumulation in microbial systems. In this study, *Bacillus* sp. HLI02 demonstrated optimal growth and PHBV production at pH 7.0, 37 °C, and a xylose concentration of 2% under a C/N ratio of 40 (Figure 4). These conditions likely reflect the strain’s natural physiological preference for balanced enzymatic activity, metabolic flux, and energy utilization, supporting efficient polymer biosynthesis.

Polymer granules act as intracellular carbonosomes that serve as energy reserves under nutrient-limited conditions. Nutrient stress, particularly nitrogen limitation coupled with excess carbon, is known to trigger PHA accumulation in *Bacillus* species [45,46,47]. The optimized C/N ratio of 40 ensured that carbon from pentose-rich sugarcane bagasse hydrolysate was effectively channelled toward PHBV synthesis rather than biomass production. Comparable trends have been reported for *Bacillus megaterium*, which accumulated up to 60% of its cell dry weight as PHB under nitrogen-limited conditions with glucose as the carbon source [48,49]. Similarly, *Bacillus flexus* produced 3.9 g/L PHB from rice bran and banana peel hydrolysate under optimized C/N ratios, highlighting the importance of carbon-to-nitrogen balance in agro-waste vaporization [50].

Temperature and pH are also critical determinants of enzymatic activity and metabolic efficiency. The preference of *Bacillus* sp. HLI02 for 37 °C aligns with the mesophilic nature of most *Bacillus* species, ensuring optimal growth rates and polymer-synthesizing enzyme activity. Neutral pH conditions stabilize intracellular enzyme systems, enhance sugar uptake, and prevent inhibitory effects that could otherwise reduce polymer accumulation. For example, *Bacillus mycoides* ICRI89 exhibited maximum PHB production at 30–37 °C and pH 7.0 when cultivated on enzymatically hydrolysed cardboard waste [51], supporting the relevance of the optimized conditions in the present study.

In this study, a xylose concentration of 2% (*w*/*v*) enabled efficient conversion of pentose sugars from sugarcane bagasse hydrolysate into PHBV. In the current study, xylose was given priority in order to highlight pentose-based bioconversion, notwithstanding *Bacillus* sp. The absence of significant inhibition during fermentation indicates that residual cellulose-derived sugars and lignin-derived phenolics were present below inhibitory thresholds. This can be attributed to the use of mild pretreatment severity and over-liming detoxification, which effectively reduce furfural, hydroxymethylfurfural (HMF), and soluble lignin fragments. The sustained cell growth, stable polymer accumulation, and high molecular weight of the extracted PHBV further confirm that hydrolysate-derived impurities did not negatively interfere with microbial metabolism or polymer biosynthesis. *Bacillus* sp. HLI02’s capacity to metabolize a wide variety of C5 and C6 sugars. While effective use of pentose sugars continues to be a significant bottleneck in lignocellulosic biorefineries, the majority of published PHA production research relies on glucose or other hexose carbohydrates. Therefore, the use of xylose as the only carbon source underlines the strain’s metabolic potential to overcome carbon catabolite preference and illustrates its appropriateness for direct conversion of hemicellulose-derived hydrolysates without the requirement for sugar supplementation or detoxification.

Pentose utilization is often a limiting factor in lignocellulosic feedstock fermentation, as many PHA-producing bacteria preferentially metabolize hexoses like glucose. Prior studies using *Bacillus cereus* S356 and *Bacillus safensis* EBT1 reported only moderate PHB yields (50–60% CDW) when utilizing sugarcane bagasse hydrolysates with mixed sugar profiles [29,52,53], highlighting the novelty of *Bacillus* sp. HLI02’s efficient pentose-to-PHBV conversion.

Overall, these findings highlight the importance of strain-specific optimization, where careful tuning of pH, temperature, carbon source concentration, and nutrient limitation can significantly enhance polymer yield [21]. It is important to highlight that the optimization performed in this work was based on an OFAT technique, which largely assesses the individual effects of process variables such as pH, temperature, and carbon concentration. While this strategy is extensively utilized for initial screening and strain characterization, it does not examine probable interactions among parameters. Nevertheless, the OFAT-based optimization successfully boosted PHBV accumulation and permitted identification of favourable physiological conditions for *Bacillus* sp. HLI02. The optimal conditions produced here provide a stable baseline for additional multivariate optimization studies. The optimized parameter set not only maximizes PHBV accumulation in *Bacillus* sp. HLI02 but also represents a foundation for scaling up the process in a cost-effective and sustainable manner, reinforcing its potential for bio-based polymer production and sustainable packaging applications.

To further test the performance of the current system, a quantitative comparison with previously reported PHBV-producing *Bacillus* strains was conducted. Carbon source, polymer yield, cellular polymer content, molecular weight, and biodegradation behaviour are among the important fermentation and polymer properties that are compiled in stat.

As shown in Table 4, *Bacillus* sp. HLI02 exhibited PHBV production efficiency comparable to or exceeding that of several previously reported *Bacillus* strains cultivated on lignocellulosic substrates. While many studies predominantly report PHB synthesis from glucose or mixed sugars, the present system demonstrates effective conversion of pentose-rich sugarcane bagasse hydrolysate into PHBV without external valerate supplementation. The polymer yield, cellular polymer content, molecular weight, and rapid biodegradation profile observed in this study highlight the metabolic robustness of *Bacillus* sp. HLI02. Notably, complete biodegradation within 60 days compares favourably with previously reported degradation periods ranging from 54 to 90 days, underscoring the environmental compatibility of the produced polymer. These results collectively demonstrate the competitiveness and industrial relevance of the proposed bioprocess for sustainable PHBV production. This comparison demonstrates *Bacillus* sp. HLI02’s competitive performance, especially its quick biodegradation profile and effective use of pentose-rich sugarcane bagasse hydrolysate.

Table 4 illustrates that the PHBV production efficiency of *Bacillus* sp. HLI02 is equivalent to or surpasses that of numerous previously documented *Bacillus* strains, especially those employing lignocellulosic substrates. The current system exhibits effective conversion of pentose sugars, substantial polymer build-up, and total biodegradation within 60 days, underscoring the innovation and industrial significance of the proposed bioprocess.

## 4. Discussion

### 4.1. Structural and Physicochemical Characterization of the Extracted PHBV Polymer

Controlled sodium hypochlorite digestion was used to remove non-PHA cellular components. This was followed by successive washings with distilled water, acetone, methanol, and diethyl ether. This process has been shown to effectively remove proteins and lipids while maintaining polymer integrity when oxidant concentration and exposure duration are properly controlled. However, excessive hypochlorite exposure may induce oxidative chain scission, resulting in reduced molecular weight, broader polydispersity, and altered thermal properties.

In the present study, the comparatively high molecular weight and modest polydispersity index imply minimal oxidative degradation during extraction. The presence of a single dominant degradation event in TGA further indicates minimal contamination by low-molecular-weight residues. Additionally, the strong ester carbonyl band in FTIR spectra and the absence of peaks associated with proteins, polysaccharides, or lipids facilitate successful polymer separation.

Although FTIR and NMR exhibited similar structural identity as compared to commercial PHBV, this confirms the purity of the produced PHBV polymer. However, this study showed good thermal stability, semi-crystalline structure, biodegradability behaviour, and outstanding cytocompatibility, which confirms that any residual contaminants are still below levels that have an impact on material performance. In order to further improve polymer quality and scalability, future research will concentrate on quantitative purity assessment and alternative extraction strategies, such as enzymatic and supercritical CO_2_-based approaches. The applied extraction method is therefore appropriate for packaging and non-implantable applications.

### 4.2. Nuclear Magnetic Resonance (NMR)

**Figure 5:**
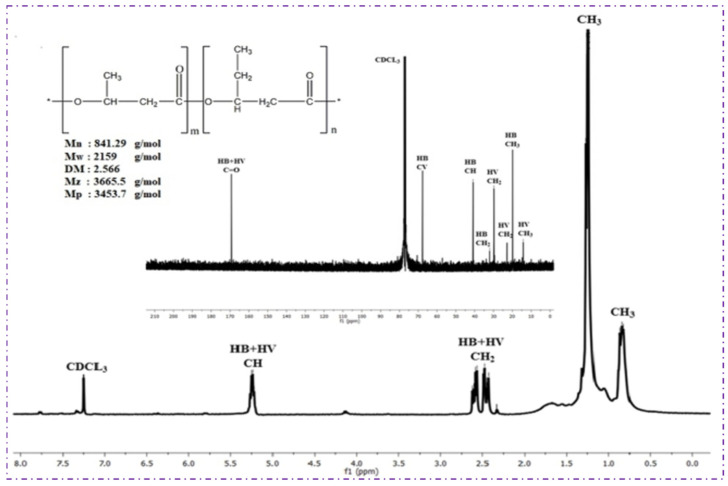
^1^H and ^13^C NMR Spectra of PHBV polymer extracted from *Bacillus* sp. HLI02.

The ^1^H and ^13^C NMR spectra confirmed the successful biosynthesis of poly(3-hydroxybutyrate-co-3-hydroxyvalerate) (PHBV) by *Bacillus* sp. HLI02 from sugarcane bagasse hydrolysate (Figure 5). In the ^1^H NMR spectrum, chemical shifts between 0.86 and 0.89 ppm correspond to the terminal methyl groups of the 3-hydroxyvalerate (3HV) monomer, while the doublets at 1.29–1.33 ppm indicate methyl protons from both HB and HV units. The multiplets observed at 1.58–1.88 ppm and 2.49–2.63 ppm correspond to methylene protons in the HV and HB chains, and the signal at 5.18–5.38 ppm represents the methine proton (–CH) adjacent to the ester linkage. These chemical shifts are consistent with previously reported PHBV spectra, confirming the copolymer composition and incorporation of valerate monomers [54].

The ^13^C NMR spectrum further supported the copolymer structure (Figure 5). The peak at 169.21 ppm corresponds to the carbonyl (C=O) carbon of the ester group, while signals at 67.72, 40.65, 29.34, and 19.37 ppm are attributable to methine, methylene, ethyl, and methyl carbons of the HB and HV units, respectively. The presence of these characteristic carbon signals confirms the successful incorporation of HV units into the polymer chain, resulting in PHBV rather than pure PHB. The signal at 77 ppm corresponds to the deuterated solvent (CDCl_3_).

Structurally, the incorporation of HV units in PHBV introduces irregularity in the polymer chain, reducing crystallinity relative to PHB and enhancing flexibility, mechanical strength, and processability. This structural characteristic is particularly significant given that sugarcane bagasse hydrolysate is rich in pentose sugars, which are generally challenging to convert efficiently into HV-containing copolymers [55,56]. The NMR analysis thus not only confirms polymer identity but also supports the novelty of this study, namely, efficient PHBV production from pentose-rich agro-industrial residues using a newly isolated *Bacillus* strain.

In comparison with other *Bacillus*-derived PHBV or PHB reported in the literature, in this study, PHBV shows distinct HV incorporation patterns, indicating that *Bacillus* sp. HLI02 is highly effective in converting mixed sugars from lignocellulosic feedstocks into value-added copolymers suitable for sustainable packaging and biomedical applications.

### 4.3. Fourier Transform Infrared Spectroscopy (FTIR)

**Figure 6:**
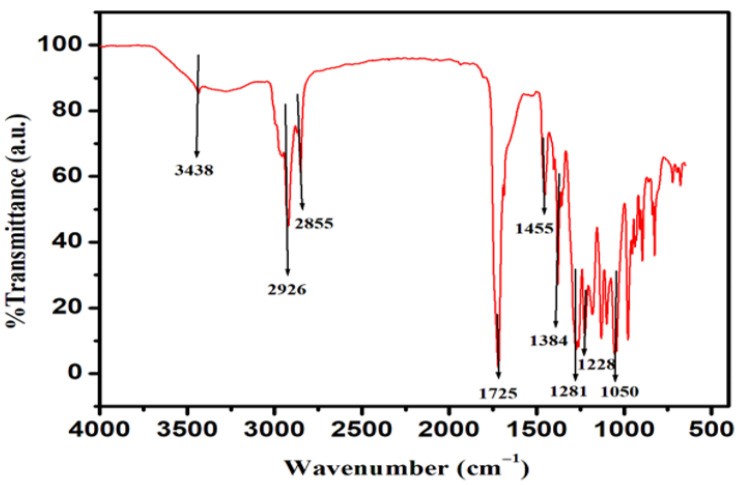
FTIR Spectra of PHBV polymer extracted from *Bacillus* sp. HLI02.

FTIR was employed to confirm the functional groups and chemical structure of the extracted polymer (Figure 6). The prominent absorption band observed at 1725 cm^−1^ corresponds to the ester carbonyl (C=O) stretching vibration, a characteristic signature of polyhydroxyalkanoates and a key indicator of polyester formation. This strong and sharp peak suggests the presence of well-ordered crystalline regions within the polymer matrix, which is typical of PHBV and is consistent with previously reported spectra [57]. Another notable peak at 1281 cm^−1^ is attributed to the C–O–C stretching vibration of the ester linkage, further confirming the polyester backbone of the polymer. The presence of these ester-related vibrations demonstrates the successful biosynthesis of PHBV rather than non-polymeric lipid residues or incomplete degradation products. Similar FTIR absorption patterns have been reported for PHBV produced by *Bacillus* species and other microbial systems [58,59], reinforcing the structural consistency of the polymer obtained in this study.

The identification of these functional groups corroborates the NMR findings, collectively confirming the copolymeric nature of the material with intact ester bonds essential for mechanical integrity and biodegradability. The preservation of ester functionalities is particularly important for applications in sustainable packaging, as these groups govern polymer processability, thermal behaviour, and susceptibility to hydrolytic and microbial degradation.

### 4.4. X-Ray Diffraction (XRD) Analysis

**Figure 7:**
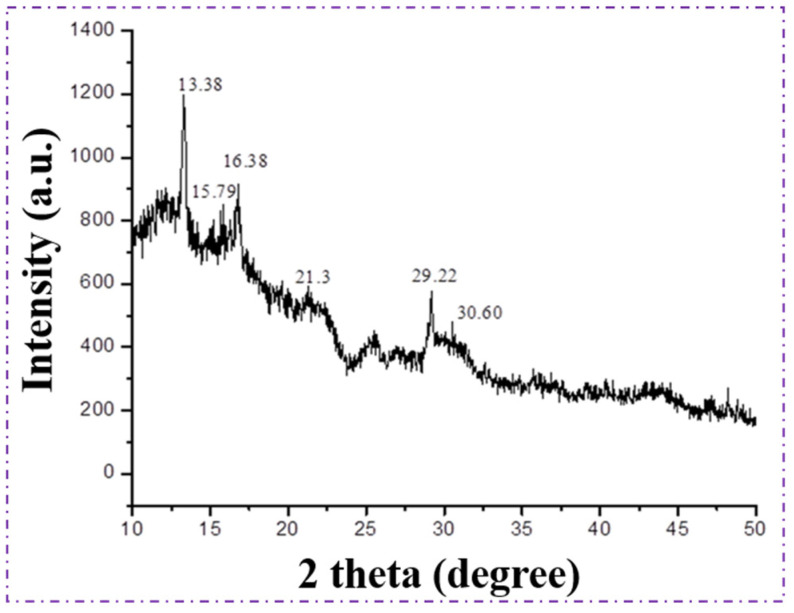
XRD Spectra of PHBV polymer extracted from *Bacillus* sp. HLI02.

The crystalline structure of the extracted polymer was examined using X-ray diffraction (XRD), and the resulting diffraction pattern is presented in Figure 7. The polymer produced by *Bacillus* sp. HLI02 exhibited prominent diffraction peaks at 2θ values of 13.38°, 16.38°, 21.30°, 25.03°, and 29.22°, which correspond to the (020), (110), (101), (121), and (002) lattice planes, respectively. These reflections are characteristic of the orthorhombic crystalline structure commonly reported for polyhydroxyalkanoates, particularly PHB and PHBV, confirming the semicrystalline nature of the synthesized polymer [60,61].

Compared to the diffraction pattern of standard PHB, notable peak shifts and a reduction in peak intensity were observed. These changes indicate alterations in crystal packing and lattice regularity, which are typically associated with the incorporation of 3-hydroxyvalerate (3HV) units into the PHB backbone. The broadening of diffraction peaks further suggests a decrease in overall crystallinity, as well as a reduction in crystallite size. Such structural modifications are advantageous, as lower crystallinity generally translates into improved flexibility, toughness, and processability—properties that are highly desirable for sustainable packaging materials [62]. The diffraction profile of the extracted PHBV closely resembles that of PHB homopolymer, which can be attributed to the relatively low molar fraction of 3HV units in the copolymer. At low 3HV contents, the crystal structure of PHBV remains largely PHB-like, with only subtle disruptions in the crystalline lattice. Similar XRD patterns and crystallinity trends have been reported in earlier studies investigating PHBV produced by microbial fermentation, particularly when the 3HV content is limited [63]. Overall, the XRD analysis confirms that the polymer synthesized from sugarcane bagasse hydrolysate possesses a semi-crystalline PHBV structure with reduced crystallinity compared to PHB. This structural feature supports its potential application in flexible packaging and biodegradable products, where a balance between mechanical strength and ductility is required. The observed crystallinity modulation further demonstrates the effectiveness of the bioprocess in tailoring polymer properties through controlled microbial synthesis.

### 4.5. Thermogravimetric Analysis (TGA)

**Figure 8:**
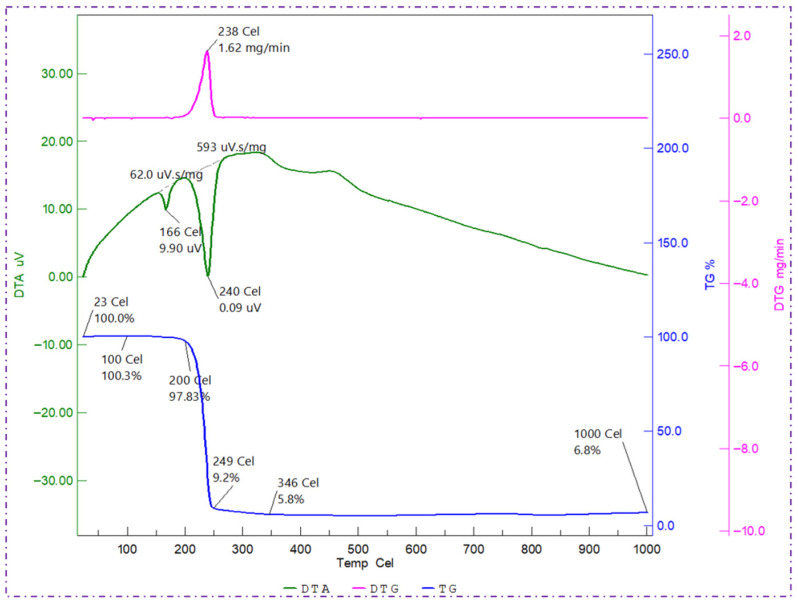
Thermal degradation Pattern of PHBV polymer from *Bacillus* sp. HLI02.

The thermal behaviour of the PHBV polymer produced by *Bacillus* sp. HLI02 was evaluated using thermogravimetric analysis (TGA), derivative thermogravimetry (DTG), and differential thermal analysis (DTA), and the results are presented in Figure 8. The DTG curve revealed a major degradation event with a maximum decomposition temperature (Td) of approximately 238 °C, indicating the thermal breakdown of the polyester backbone. The presence of a single dominant degradation peak suggests a relatively homogeneous polymer system, while the breadth of the peak reflects the copolymeric nature of PHBV, composed of both 3-hydroxybutyrate (3HB) and 3-hydroxyvalerate (3HV) units.

The DTA thermogram exhibited two distinct and sharp endothermic peaks during heating. The first peak, observed at 166 °C, corresponds to the melting temperature (Tm) of the PHBV polymer. This melting point is lower than that typically reported for PHB homopolymer (~175–180 °C), which is consistent with the incorporation of 3HV units that disrupt the crystalline packing of PHB and reduce melting temperature. Such melting behaviour confirms the formation of a PHBV copolymer and aligns well with previously reported thermal profiles of microbial PHBV [64,65]. The second DTA peak is attributed to crystal reorganization or recrystallization phenomena occurring during heating, a behaviour commonly observed in semi-crystalline polymers.

The TGA curve further provides insight into the thermal stability of the polymer. The onset degradation temperature (Ti), defined as the temperature at which 5% weight loss occurs, was observed at approximately 200 °C, while the complete degradation temperature (Tc), corresponding to 95% weight loss, occurred at around 346 °C. These values indicate a relatively wide thermal processing window and demonstrate that the PHBV produced by *Bacillus* sp. HLI02 possesses high thermal stability. Such stability is essential for melt processing techniques, including extrusion and film casting, which are commonly employed in packaging applications.

Overall, the combined TGA, DTG, and DTA analyses confirm that the PHBV synthesized from sugarcane bagasse hydrolysate exhibits favourable thermal characteristics, including a reduced melting temperature, enhanced processability, and good thermal resistance [66]. These properties make the polymer particularly suitable for sustainable packaging applications, where thermal stability, mechanical performance, and biodegradability must be carefully balanced.

### 4.6. Gas Permeation Chromatography (GPC)

**Figure 9:**
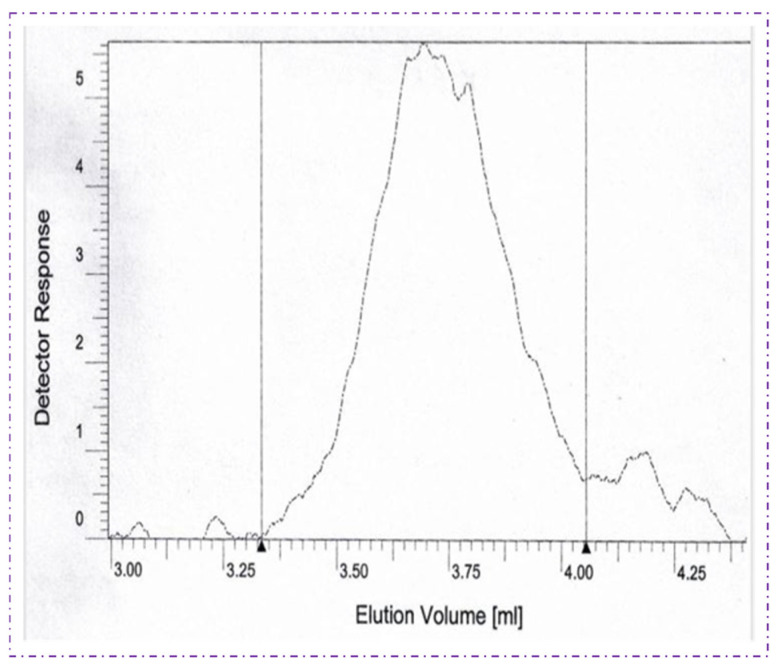
GPC spectra of PHBV polymers extracted from *Bacillus* sp. HLI02.

The molecular weight characteristics of the PHBV polymer produced by *Bacillus* sp. HLI02 was determined by gel permeation chromatography (GPC), as shown in Figure 9. The analysis revealed the number-average molecular weight (Mn) and weight-average molecular weight (Mw), from which the polydispersity index (PDI = Mw/Mn) was calculated. The PHBV polymer exhibited a PDI value of 2.56, indicating a moderately narrow molecular weight distribution typical of biologically synthesized polyhydroxyalkanoates. This PDI value is relatively lower than that reported for PHA produced by *Bacillus* sp. INT005 using glucose as a carbon source, where broader molecular weight distributions were observed [67]. The comparatively narrower distribution obtained in the present study suggests a more controlled polymer chain growth during biosynthesis, which may be attributed to the optimized fermentation conditions and the utilization of pentose-rich sugarcane bagasse hydrolysate. Controlled molecular weight distribution is advantageous, as it enhances polymer processability, mechanical consistency, and reproducibility during material fabrication. The molecular weight profile also reflects the metabolic efficiency of *Bacillus* sp. HLI02 in channelling carbon flux toward polymer elongation rather than premature chain termination. PHBV polymers with moderate-to-high molecular weights and balanced polydispersity are particularly desirable for packaging applications, as they offer a favourable combination of tensile strength, flexibility, and melt-processing stability. Overall, the GPC results demonstrate that the PHBV synthesized in this study possesses molecular weight characteristics comparable to, or better than, those reported for PHAs produced from conventional sugar substrates.

### 4.7. Surface Characterization of Polymer

**Figure 10:**
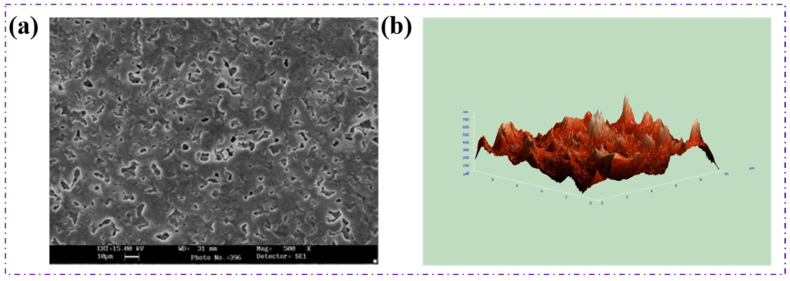
(**a**) SEM image of PHBV film (**b**) Surface study of PHBV film by Scanning Probe Microscopy (SPM).

The surface morphology and microstructural features of the PHBV thin film were analyzed using scanning probe microscopy (SPM), as shown in Figure 10. The PHBV film was prepared by solvent casting from a chloroform solution, followed by gradual solvent evaporation, a process known to influence crystallization behaviour and surface topology in semi-crystalline polymers.

The SEM images revealed a heterogeneous surface characterized by visible protuberances, irregular domains, and bubble-like structures, as illustrated in Figure 10a. These surface features are primarily attributed to solvent evaporation–induced crystallization, where rapid phase separation between polymer-rich and solvent-rich regions occurs during film formation. As chloroform evaporates, localized polymer aggregation and crystallite growth lead to surface roughness and the formation of protrusions. Similar morphological patterns have been reported for PHBV and PHB films prepared via solvent casting, particularly when evaporation is uncontrolled or occurs at ambient conditions [15,68]. The presence of surface irregularities and microvoids is also consistent with the semi-crystalline nature of PHBV, as confirmed by XRD analysis. The incorporation of 3-hydroxyvalerate (3HV) units disrupts the regular packing of PHB chains, leading to non-uniform crystallization and increased amorphous regions. This morphological heterogeneity is often associated with enhanced flexibility and reduced brittleness, which are desirable properties for packaging films.

SPM analysis further supports these observations by revealing nanoscale variations in surface roughness, indicating a crystallite distribution across the film surface. While such roughness may influence optical clarity, it can be advantageous for applications requiring improved interfacial adhesion, such as coatings, multilayer packaging structures, or biomedical scaffolds.

### 4.8. Biocompatibility of Polymer

**Figure 11:**
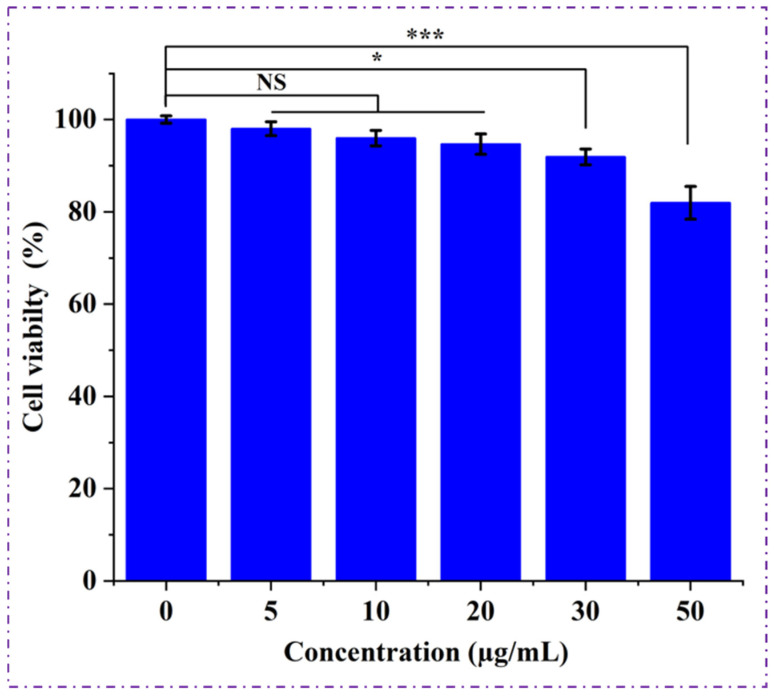
MTT Assay for cell viability of PHBV polymer extracted from *Bacillus* sp. HLI02 NIH mouse fibroblast cell line (NS—Not Significant, *—Significant, ***—Very higliy significant).

The in vitro biocompatibility of the extracted PHBV polymer was evaluated using the NIH/3T3 mouse fibroblast cell line through an MTT assay, and the results are presented in Figure 11. Cell viability analysis across different polymer concentrations revealed no significant reduction in metabolic activity, indicating that the PHBV films did not exert cytotoxic effects on fibroblast cells. Therefore, even at the highest tested concentration of 50 µg/mL, cell viability remained comparable to the control, demonstrating excellent cyto-compatibility of the polymer. A marginal reduction in cell viability was observed at the highest polymer concentration (50 µg/mL). Similar concentration-dependent reductions in cell viability at higher PHBV coating densities have been reported in previous studies and are generally attributed to surface coverage effects rather than polymer-induced cytotoxicity [7,23]. This decrease does not indicate cytotoxicity but is attributed to a concentration-dependent physical effect commonly reported for hydrophobic polymer films. At elevated coating densities, polymer layers can partially restrict cell–substrate interactions, reduce nutrient diffusion, and interfere with mitochondrial dye uptake during MTT analysis. Similar dose-dependent reductions at higher PHBV concentrations have been widely reported and are associated with surface coverage effects rather than polymer-induced cellular toxicity. Importantly, cell viability at all tested concentrations remained above the 70% threshold defined by ISO 10993-5 (DIN EN ISO 10993-5), confirming the non-cytotoxic and biocompatible nature of the synthesized PHBV.

The consistent viability trend observed across all tested concentrations suggests that the PHBV produced by *Bacillus* sp. HLI02 does not release toxic residues or harmful degradation products under in vitro conditions. This non-cytotoxic behaviour is a well-recognized characteristic of PHAs in general and is attributed to their natural metabolic origin and the absence of harmful additives or catalysts commonly associated with petrochemical polymers. Similar biocompatibility profiles have been reported for PHBV synthesized by microbial fermentation, where fibroblast and osteoblast cells exhibited normal proliferation and attachment on PHBV substrates [69].

The observed biocompatibility further supports evidence for the structural integrity of the polymer, as confirmed by FTIR and NMR analyses, and supports its suitability for applications requiring direct or indirect biological contact. While the primary focus of this study is on sustainable polymer production from agro-industrial residues, the demonstrated cytocompatibility broadens the potential application spectrum of the synthesized PHBV to include biomedical materials, food-contact packaging, and agricultural films.

### 4.9. Biodegradability of Polymer

**Figure 12:**
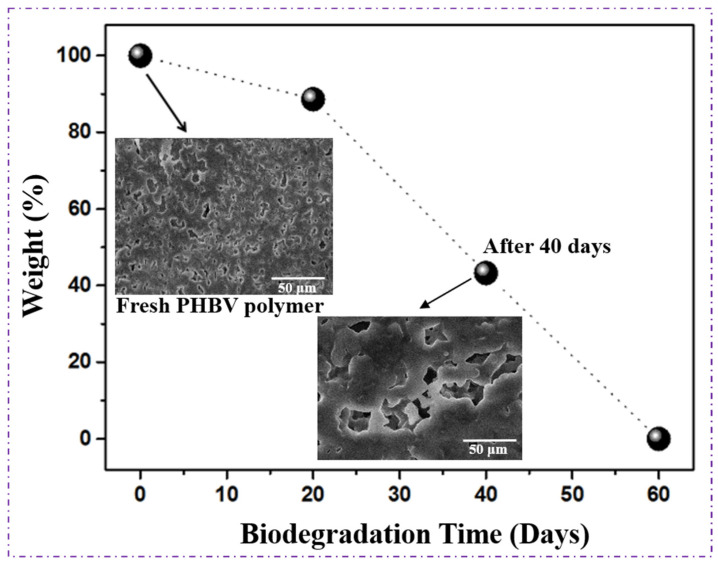
The biodegradation weight loss of the polymer is a function of time. The inset microscopic images captured by SEM explicitly revealed the material loss by evolution of holes and cavities in the PHBV polymer extracted from *Bacillus* sp. HLI02.

The biodegradability of the PHBV produced by *Bacillus* sp. HLI02 was evaluated using an open-window composting method by burying a known weight of polymer in soil and monitoring weight loss and morphological changes at 20, 40, and 60 days. As illustrated in Figure 12, the PHBV samples exhibited a gradual and continuous decrease in weight over time, achieving complete degradation within 60 days. This progressive mass loss confirms the high susceptibility of the polymer to microbial and enzymatic attack under natural soil conditions.

Morphological examination of the degrading PHBV using SPM further supported these observations. The SEM images revealed the progressive formation of surface pores, cracks, and cavities with increasing composting time, indicating polymer chain scission and material erosion due to microbial activity in Figure 12 after 40 days. Such surface deterioration is characteristic of polymer degradation, where extracellular depolymerases secreted by soil microorganisms initiate hydrolytic cleavage of ester bonds, ultimately leading to complete mineralization of the polymer [70].

Comparable biodegradation behaviour has been reported for PHAs synthesized by other *Bacillus* species. For instance, PHAs produced by *Bacillus endophyticus* were completely degraded within 54 days under similar open-window composting conditions [44]. The slightly longer degradation period observed in the present study may be attributed to differences in polymer composition, crystallinity, molecular weight, or valerate content, all of which are known to influence degradation kinetics. Nonetheless, the complete degradation of PHBV within 60 days demonstrates its excellent environmental compatibility and reinforces its suitability for applications requiring rapid end-of-life biodegradation, such as sustainable packaging, biomedical and even more agricultural materials [70]. The high biodegradation rate observed in this study may also be facilitated by the low crystallinity and pentose-derived monomer incorporation from sugarcane bagasse hydrolysate, which enhances microbial accessibility.

The observed degradation rate aligns with the requirements of international standards for compostable packaging. The European standard EN 13432 (European Committee for Standardization (CEN)) requires that packaging materials must achieve at least 90% biodegradation within 6 months under industrial composting conditions [71], whereas the U.S. standard ASTM D6400 (ASTM International) requires complete disintegration and at least 90% conversion of carbon to CO_2_ within a defined timeframe [72]. The complete biodegradation of PHBV from *Bacillus* sp. HLI02 within 60 days demonstrates that the polymer meets and even surpasses these thresholds, highlighting its potential suitability for industrially compostable packaging applications [73].

Overall, these results indicate that the PHBV polymer synthesized from sugarcane bagasse is not only biodegradable under natural conditions but also aligns with recognized international standards for compostable materials. This supports its application as a sustainable alternative to conventional petrochemical-based plastics in packaging, where rapid end-of-life degradation is desirable to reduce environmental impact. Although the present study demonstrates successful PHBV biosynthesis from sugarcane bagasse hydrolysate and provides comprehensive physicochemical, thermal, biodegradation, and biocompatibility characterization, mechanical properties such as tensile strength, elongation at break, and Young’s modulus were not evaluated. The practicality of biopolymers in packaging systems, where processability and end-use performance are determined by mechanical integrity and flexibility, depends on these factors. Therefore, one of the study’s limitations is the lack of mechanical testing. Nevertheless, the discovered molecular weight distribution, reduced crystallinity, integration of 3-hydroxyvalerate units, and excellent thermal stability strongly suggest mechanical behaviour akin to previously described PHBV materials suited for packing applications. Future research will concentrate on material reinforcement techniques to customize the strength–ductility balance for particular packaging requirements, as well as on extensive mechanical characterization utilizing standardized tensile testing methods (ASTM D882 or ISO 527).

## 5. Conclusions and Future Prospective

This study presents a novel, integrated bioprocess for the sustainable production of poly(3-hydroxybutyrate-co-3-hydroxyvalerate) (PHBV) using sugarcane bagasse hydrolysate as a low-cost carbon source and *Bacillus* sp. and HLI02 as a robust microbial producer. Optimization of key parameters, including pH, temperature, C/N ratio, and pentose concentration, enabled enhanced polymer accumulation, demonstrating the strain’s effective conversion of lignocellulosic sugars into a high-value copolymer. Under optimized fermentation conditions, the strain efficiently converts the hemicellulose sugars into PHBV, yielding 2 g/L polymer corresponding to 59.5% CDW, without the requirement of additional supplementation for PHBV production. Comprehensive polymer characterization confirmed the formation of a structure pure PHBV polymer with a weight average molecular weight of 2.16 kDa, moderate polydispersity (2.56), and a semi-crystalline structure. Thermal analysis revealed a melting temperature of 166 °C and suitability for melt processing applications. The extracted PHBV was non-cytotoxic, as confirmed by in vitro fibroblast cell assays, and exhibited complete biodegradation within 60 days under soil composting conditions, proving its potential as a compostable polymer in alignment with industrial standards (EN 13432/ASTM D6400). All of these findings demonstrate *Bacillus* sp. HLI02 has a potent metabolic capacity to effectively use C5 sugars and get beyond one of the main obstacles in lignocellulosic biorefineries. The study offers convincing proof that sugarcane bagasse hydrolysate can be used as a low-cost, efficient substrate for PHBV production, allowing for the simultaneous synthesis of bioplastics and waste valorization. Therefore, Pentose-rich agro-industrial residues, traditionally underutilized in biopolymer production, can be directly converted into high-performance PHBV using robust *Bacillus* strains, offering a scalable, environmentally benign, and economically viable route toward next-generation sustainable packaging materials. Overall, this study demonstrates an environmentally friendly platform for producing high-performance bio-based polymers from agro-industrial residues. The integrated approach not only addresses polymer yield and quality but also emphasizes upstream and downstream sustainability, providing a roadmap for future scale-up and industrial implementation.

Although the current study demonstrated the effective production of PHBV from pentose-rich sugarcane bagasse hydrolysate, the quantitative analysis of minor sugars such as arabinose and trace inhibitors is beyond the scope of this current study. Future studies will be directed toward the comprehensive analysis of the composition of the hydrolysate and the fermentation kinetics of mixed sugars to further clarify the distribution of the carbon source in the biosynthesis of PHBV. Apart from pH and temperature, other important parameters for PHBV production include inoculum size, agitation speed, incubation time, and other carbon sources. These parameters are known for affecting PHBV production and the oxygen transfer rate during the microbial fermentation process. Future studies will focus on the multivariable optimization of PHBV production using statistical tools such as factorial experimental design and response surface methodology (RSM).

Future investigations will include tensile strength, elongation at break, and Young’s modulus measurements to comprehensively assess the suitability of the material for packaging applications.

## Figures and Tables

**Table 1:** Composition of MSM Production Media.

Ingredients	Concentration
NaCl	10.0 g/L
KH_2_PO_4_	0.5 g/L
K_2_HPO_4_	0.5 g/L
Malic acid	2.7 g/L
Glutamic acid	1.5 g/L
Yeast extract	4 g/L
(NH_4_)_2_ SO_4_	2.38 g/L
Sugar (Xylose)	Varying concentration range of xylose 1–5 g/L

**Table 2:** Sugar Utilization Test.

Sugar Utilization Test	*Bacillus* sp. HLI02
Lactose	−
Xylose	+
Maltose	+
Fructose	+
Dextrose	+
Galactose	+
Raffinose	+
Trehalose	+
Melibiose	+

+: Able to utilize, −: Not able to utilize.

**Table 3:** Biochemical Data.

Biochemical Test	*Bacillus* sp. HLI02
Growth at 10% NaCl	+
Hippurate hydrolysis	−
Anaerobic growth	−
MR test	−
VP test	+
Citrate Reductase	−
Starch Hydrolysis	+
Oxidase reductase	+
Casein hydrolysis	−
Urease hydrolysis	+
Nitrate Hydrolysis	+
Esculin Hydrolysis	−
Growth at 45 °C	+
Catalase	+
H_2_S production	−
Indole	−
Ammonia production	−
Citrate utilization	+
Mannitol	+
Esculin hydrolysis	+
Anaerobic growth	−
Blood Haemolysis	−
Gelatinase	+
Casein hydrolysis	+
Tributyrin	+
Lipase	+
Cellulose	+
Chitin hydrolysis	−
Pectin hydrolysis	−
DNase	+
Lecithinase	−

+: Able to produce, −: Not able to produce, MR—Methyl Red, VP—Voges Proskauer.

**Table 4:** Comparative analysis of PHBV production utilizing a bacillus strain cultured on different carbon sources.

Microorganism	Carbo Source	Polymer	Yield (g/L)	%CDW	Mw (kDa)	Biodegradation (Days)	Reference
*Bacillus cereus* S356	Sugarcane bagasse hydrolysate	PHBV	2.8	55–60	280	90	[29]
*Bacillus safensis* EBT1	Bagasse hydrolysate	PHB	3.2	58	240	75	[53]
*Bacillus flexus*	Banana peel hydrolysate	PHB	3.9	62	310	80	[50]
*Bacillus megaterium*	Glucose	PHB	4.5	60	350	-	[48]
*Bacillus endophyticus*	Mixed sugars	PHBV	3.1	65	290	54	[44]
*Bacillus* sp. HLI02	Sugarcane bagasse hydrolysate	PHBV	2	59.5	2.16	60	This study

CDW-% of cell dry weight.

## Data Availability

The original contributions presented in this study are included in the article. Further inquiries can be directed to the corresponding authors.

## Supplementary Materials

The following supporting information can be downloaded at: https://www.mdpi.com/article/10.3390/polym18070802/s1, Figure S1: Phylogenetic tree of *Bacillus* sp. HLI02 and its homologous sequences; Figure S2: Secondary structure model of 16S rRNA of *Bacillus* sp. HLI02.
