# Supplementary material for: Agro-Industrial Waste Valorization for Sustainable PHBV Production from Sugarcane Bagasse Using Bacillus sp. HLI02
Source: Polymers (Basel). 2026 Mar 26;18(7):802. doi: 10.3390/polym18070802 (PMC13075205; doi:10.3390/polym18070802)
Supplement: Supplementary file 1 [file polymers-18-00802-s001.zip › polymers-4123247-supplementary.pdf]

Supplementary Materials

Agro-Industrial Waste Valorization for Sustainable PHBV Production from Sugarcane Bagasse Using *Bacillus* sp. HLI02

Komal Singh [1,†], Preeti Tomer [1,†], Debarati Paul [2], Narayan Chandra Mishra [3], Tanushri Mukherjee [2], Debashish Ghosh [4], Monica Trif [5,†], Sourish Bhattacharya[2], Alexandru Vasile Rusu [6,*,†] and Saugata Hazra [1,7,*]

[1] Department of Bioscience & Bioengineering, Indian Institute of Technology, Roorkee 247667, India; komal_s@pe.iitr.ac.in (K.S.); preetitomer7@gmail.com (P.T.)
[2] CSIR-Central Salt and Marine Chemicals Research Institute, Bhavnagar 364002, India; pauldebarati27@gmail.com (D.P.); tanumukherji06@gmail.com (T.M.); sourishb.csmcri@csir.res.in (S.B.)
[3] Department of Polymer and Process Engineering, Indian Institute of Technology, Roorkee 247667, India; narayan.mishra@pe.iitr.ac.in
[4] Material Resource Efficiency Division, CSIR-Indian Institute of Petroleum, Mohkampur, Dehradun 248005, India; debashish.ghosh@csir.res.in
[5] Department of Food Science, University of Agricultural Sciences and Veterinary Medicine Cluj-Napoca, Manastur 3–5, 400372 Cluj-Napoca, Romania; monica_trif@hotmail.com
[6] CENCIRA Agrofood Research and Innovation Centre, Ion Meșter 6, 400650 Cluj-Napoca, Romania
[7] Centre for Nanotechnology, Indian Institute of Technology Roorkee, Roorkee 247667, India
* Correspondence: author: rusu_alexandru@hotmail.com (A.V.R.); saugata.hazra@bt.iitr.ac.in (S.H.)
† These authors contributed equally to this work.

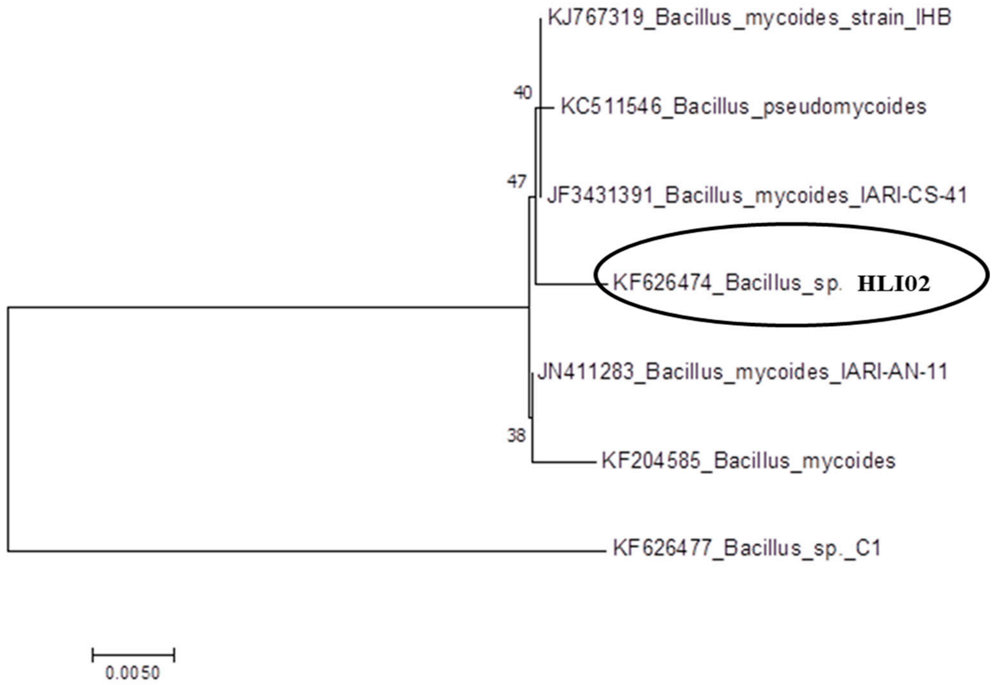

Figure S1. Phylogenetic tree of *Bacillus* sp. *HLI02* and its homologous sequences.

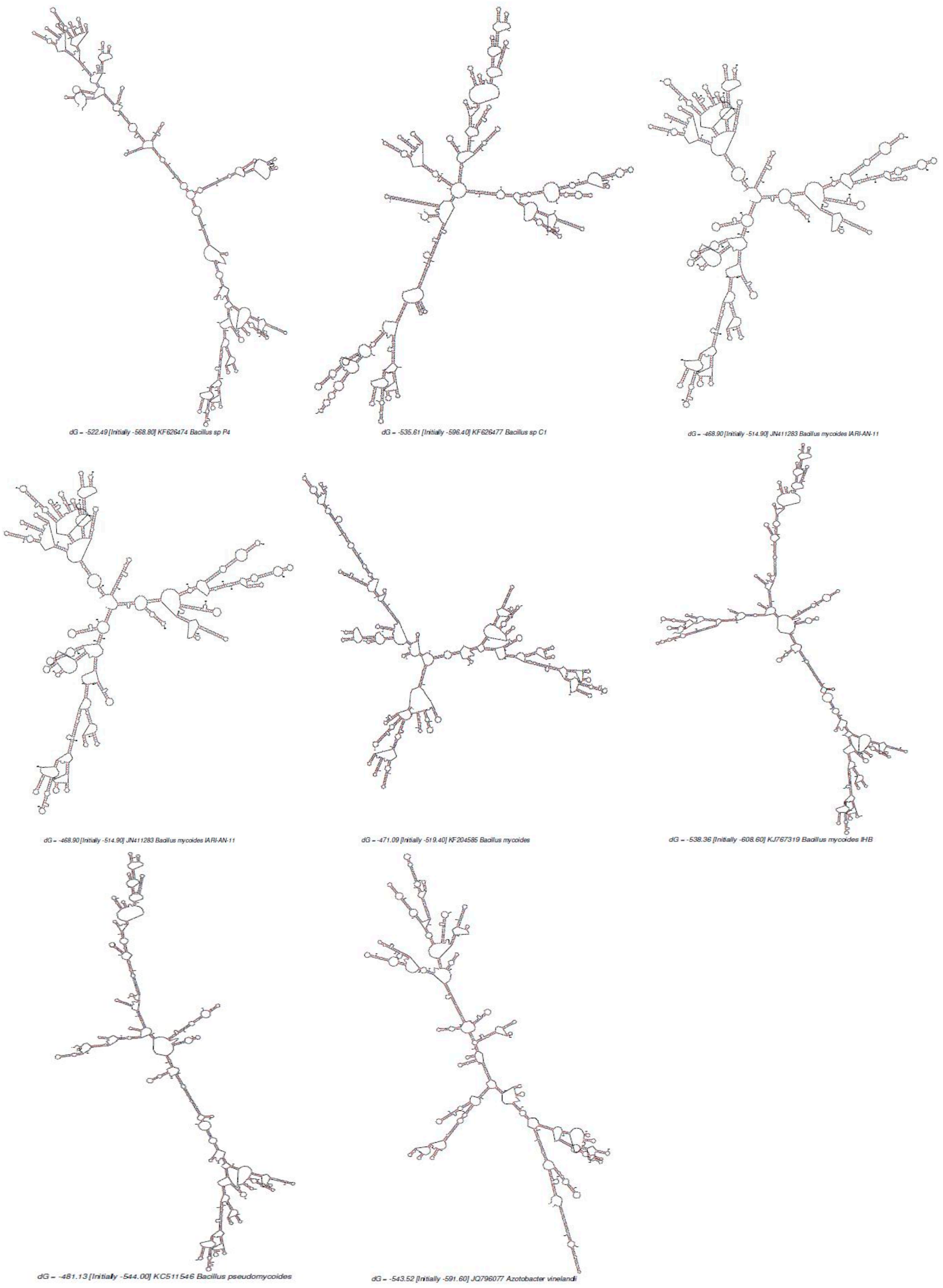

Figure S2. Secondary structure model of 16S rRNA of Bacillus sp. HLI02.
